# Inequities in Neuropsychiatric Outcomes After Brain Trauma in the All of Us Database

**DOI:** 10.1001/jamanetworkopen.2025.39313

**Published:** 2025-10-24

**Authors:** Tadeusz H. Wroblewski, Favour C. Ononogbu-Uche, Pemla Jagtiani, Rose M. E. Calixte, Marie-Claire Roberts, Peter B. Barr, Tim B. Bigdeli, Ernest J. Barthélemy

**Affiliations:** 1 College of Medicine, SUNY Downstate Health Sciences University, Brooklyn, New York; 2 Global Neurosurgery Laboratory, SUNY Downstate Health Sciences University, Brooklyn, New York; 3 Institute for Genomics in Health, SUNY Downstate Health Sciences University, Brooklyn, New York; 4 MD-PhD Program, SUNY Downstate Health Sciences University, Brooklyn, New York; 5 Department of Epidemiology and Biostatistics, School of Public Health, SUNY Downstate Health Sciences University, Brooklyn, New York; 6 College of Nursing, SUNY Downstate Health Sciences University, Brooklyn, New York; 7 Department Community Health Sciences, School of Public Health, SUNY Downstate Health Sciences University, Brooklyn, New York; 8 Department of Psychiatry and Behavioral Sciences; SUNY Downstate Health Sciences University, Brooklyn, New York; 9 VA New York Harbor Healthcare System, Brooklyn, New York; 10 Department of Surgery, One Brooklyn Health/Brookdale University Hospital and Medical Center, Brooklyn, New York; 11 Department of Neurology, SUNY Downstate Health Sciences University, Brooklyn, New York; 12 Department of Surgery, SUNY Downstate Health Sciences University, Brooklyn, New York

## Abstract

**Importance:**

Traumatic brain injury (TBI) is an important public health concern, with disparities in post-TBI neuropsychiatric diagnoses (NPDs) observed across Black or African American and White populations.

**Objective:**

To examine the incidence of NPDs following TBI in the US and assess how socioecologic factors are associated with population-level differences.

**Design, Setting, and Participants:**

This retrospective cohort study used data from the All of Us Research Program, version 7, including available electronic health record data from participants aged 18 years or older enrolled between May 6, 2018, and July 1, 2022, with an established TBI diagnosis based on diagnostic codes and criteria from the Centers for Disease Control and Prevention, Department of Defense, and clinical expertise. Data were analyzed between June 24, 2024, and January 23, 2025.

**Exposure:**

Social and structural determinants of health.

**Main Outcomes and Measures:**

The primary outcomes assessed were incident NPDs following TBI grouped into 10 diagnostic clusters according to phecode categories. Competing-risk models (subdistribution hazard ratio [HR] with 95% CI) and Heckman-type selection models (β [SE]) were used.

**Results:**

The study included 8714 participants (mean [SD] age, 49.0 [17.9] years; 4700 women [54.5%]) with TBI and was limited to participants self-identifying as Black or African American (hereafter, Black) (2192 [25.2%]) or White (6522 [74.8%]) race. White participants had a lower adjusted risk of schizophrenia or other psychotic disorders (adjusted HR [AHR], 0.49 [95% CI, 0.35-0.69]), posttraumatic stress disorder (AHR, 0.67 [95% CI, 0.52-0.86]), substance use disorders (AHR, 0.51 [95% CI, 0.42-0.62]), and headache disorders (AHR, 0.78 [95% CI, 0.67-0.91]) compared with Black participants. Models accounting for selection bias revealed a lower adjusted likelihood of schizophrenia or other psychotic disorders (β [SE], −0.24 [0.07]) and headache disorders (β [SE], −0.10 [0.03]) in White participants. Higher social deprivation was associated with a lower likelihood of mood disorders (β [SE], −0.65 [0.14]), anxiety disorders (β [SE], −0.85 [0.13]), posttraumatic stress disorder (β [SE], −1.06 [0.22]), any dementia (β [SE], −1.03 [0.22]), and headache disorders (β [SE], −0.52 [0.14]) but a higher likelihood of sleep disorders (β [SE], 0.34 [0.08]).

**Conclusions and Relevance:**

This cohort study found that post-TBI NPDs varied across populations, with a higher likelihood of psychotic and headache disorders among Black All of Us participants. These findings highlight the utility of social determinants of health for better understanding disparities in post-TBI outcomes, underscoring the need for additional efforts to address these disparities.

## Key Points

**Question:**

What is the association of neuropsychiatric diagnosis incidence following traumatic brain injury (TBI) with racial disparities?

**Findings:**

In a cohort study of 8714 participants with a history of TBI in the National Institutes of Health All of Us database, White participants were less likely than Black or African American participants to be diagnosed with psychotic, posttraumatic stress, substance use, or headache disorders. Accounting for sample selection bias, White participants had a lower probability of being diagnosed with psychotic or headache disorders.

**Meaning:**

These findings suggest that there are substantial racial disparities in neuropsychiatric outcomes following TBI, underscoring the importance of accounting for psychosocial and environmental modifiers that may alter TBI outcome trajectories.

## Teaser

This cohort study examines the incidence of neuropsychiatric diagnoses following traumatic brain injury among All of Us Research Program participants and the factors associated with racial and social deprivation differences.

## Introduction

Traumatic brain injury (TBI) has been described as a silent pandemic, impacting approximately 75 million people globally and contributing to approximately 2.8 million emergency department visits, hospitalizations, and deaths in the US annually.^1,2^ Marginalized groups, including Black or African American and Hispanic populations, have poorer functional outcomes following TBI, both at hospital discharge and 1-year follow-up, compared with White populations.^3^ Underlying socioecologic factors, including structural racism, perpetuate TBI disparities and strongly influence an individual’s trajectory both preceding and after sustaining a TBI but may not be readily measurable or observed.^4,5^

Structural racism perpetuates racial disparities through societally entrenched systems that disadvantage some groups over others and result in the inequitable distribution of resources.^6,7^ The impacts of structural racism are reflected in observed health disparities, as well as by the historical exclusion of Black or African American populations from clinical and translational research agendas. In the context of TBI, the National Academies of Sciences, Engineering, and Medicine has presented the biological, psychological, sociologic, ecologic (BPSE) prism as a mechanism to describe inequities affecting marginalized populations. The BPSE prism offers a comprehensive framework to integrate the impacts of biomedical factors and social determinants of health to understand disparities in TBI treatment, recovery, and outcome.^4,8^ Incorporating the BPSE prism into TBI research may facilitate an enhanced understanding of suboptimal TBI outcomes and prognosis among socially disadvantaged groups while revealing actionable targets for achieving global health equity in neurotrauma.^4^

In this study, race-based disparities in post–TBI incident neuropsychiatric outcomes were investigated in the All of Us Research Program.^9^ We aimed to investigate TBI outcomes in alignment with the BPSE prism, with special consideration of the impact of unmeasured social determinants of health related to structural racism, through the direct comparison of outcomes between Black or African American and White individuals. Given the pervasive disparities in TBI diagnosis and outcomes between these groups, we sought to identify and adjust for nonrandom selection of participants to account for unmeasured or unobservable variables that may influence study participation and the measured outcomes.^10,11,12,13^

## Methods

### All of Us Database

This cohort study used data available from the All of Us Research Program, a national database that enrolls participants aged 18 years or older across the US with appropriate emphasis on the inclusion of historically underrepresented populations in biomedical research. Participants provide informed consent, complete surveys, and link electronic health records (EHRs) through an online portal. This study included data from participants who were enrolled between May 6, 2018, and July 1, 2022 (version 7), in accordance with the All of Us Code of Conduct and State University of New York Downstate Health Sciences University Institutional Review Board. This study followed the Strengthening the Reporting of Observational Studies in Epidemiology (STROBE) reporting guideline.

### Study Population

Patients with TBI were selected based on *International Classification of Diseases, Ninth Revision *and* International Statistical Classification of Diseases, Tenth Revision Clinical Modification* code criteria from the Centers for Disease Control and Prevention,^14^ Department of Defense,^15^ and clinical expertise of the consulting neurosurgeon (E.J.B.), as previously described.^16^ A TBI diagnosis required at least 1 EHR record and was considered as a lifetime event. Age at injury was established from the date of index TBI. All of Us does not include traditional measures of TBI severity, such as the Glasgow Coma Scale.^17^ Therefore, we used the International Classification of Diseases Programs for Injury Categorization head or face Abbreviated Injury Score (AIS),^18^ which has been previously validated and used in EHR-based head trauma studies.^19,20,21,22^ Severity of TBI was stratified as serious (AIS ≥3) and less serious (AIS <3).^16,20^

Demographic data were derived from the All of Us The Basics Survey, including self-identified race (Asian, Black or African American [hereafter, Black], Middle Eastern or North African, Native Hawaiian or Other Pacific Islander, White, multiracial, and other [none of these, none indicated, prefer not to answer, skip question]), self-reported ethnicity (Hispanic or Latino, non–Hispanic or Latino), and gender (man, woman, and other [nonbinary; transgender; none of these describe me, and I would like to consider additional options; and prefer not to answer]). Black and White race were used exclusively in the analysis because of sociohistorical determinants that drove the emergence of the terms Black and White as the predominant racialized categories of social identity for most of US history; therefore, we reasonably expected that analysis of these 2 racial categories would best capture contemporary racial disparities in TBI outcome in the US.^23^ Gender was included to account for other potential social and structural factors impacting variation in TBI outcomes across groups. Variation within the socioeconomic sphere of social determinants of health was proxied using the social deprivation index (SDI), an area-level composite measure.^24^ Additional subcomponents of the SDI were reported as descriptive characteristics but not included in regression models. The SDI quartiles were established based on all participants with available data. Comparisons across racial groups were assessed using the Pearson χ^2^ test and Welch *t* test.

### Neuropsychiatric Diagnostic Clusters

The primary outcome measures were incident neuropsychiatric diagnoses (NPDs) following the index TBI.^25,26,27,28^ Ten diagnostic clusters were derived from *International Classification of Diseases, Ninth Revision *and* International Statistical Classification of Diseases, Tenth Revision Clinical Modification* codes according to broad phecode^29^ categories (PheWAS R package^30^), as previously described.^16,31^ Following best practices, incident NPDs required the presence of 2 or more phecodes in a participant’s EHR,^31,32^ as well as no diagnosis prior to the index TBI. Time to incident NPD was measured from the index TBI to the EHR record of diagnosis. Individuals who did not have an incident NPD were censored at the time of the last EHR encounter or time of recorded death (eTable 1 in Supplement 1). Participants with EHR encounters corresponding to an NPD that could not be mapped to a phecode were excluded.

### Statistical Analysis

Data were analyzed between June 24, 2024, and January 23, 2025. For each NPD (*N_i_*), the analytic sample was established after excluding individuals with a preexisting diagnosis of condition *N* and censoring EHR diagnoses from the first 6 months following index TBI to account for direct trauma-related sequelae and undiagnosed preexisting conditions^33^ (eFigure 1 in Supplement 1). Participants with missing gender or SDI data were excluded from subsequent analyses. Rate of diagnosis is reported as the crude incidence rate (IR) (ie, diagnoses per 1000 person-years), with the 95% CI computed by exact Poisson method incorporating follow-up time as an offset. Incidence rates were compared using the likelihood ratio test.

Next, we fit competing-risk regression models (Fine-Gray method), adjusting for race, ethnicity, age at index TBI, gender, SDI, injury severity, and preexisting comorbid NPDs (when *P* < .10 in univariable models) (eTable 2 in Supplement 1). Outcomes were reported as subdistribution hazard ratios (HRs) with 95% CIs, with death as a competing risk. Sample selection bias was assessed using the Lagrange multiplier test,^34^ followed by Heckman-type selection models, which are a variant of the Heckman 2-step selection model originally developed to detect and statistically correct for selection bias in econometric models.^35,36^ We used bivariate probit models with sample selection to estimate the dichotomous presence or absence of post-TBI incident NPDs,^11,12,37,38,39,40^ modeling selection into the TBI group with a control cohort (eTable 3 and eMethods in Supplement 1). Coefficient estimates (β) with SEs are reported. Significance was determined after Bonferroni correction for multiple testing (*P* < .005). All analyses were performed in the All of Us Researcher Workbench using R, version 4.4.0 (R Foundation for Statistical Computing).

Importantly, Omar et al^23^ posited that one way racism continues to be institutionalized in research is through a persistent portrayal of Blackness as a risk factor compared with a White reference group, which is often due to convention rather than statistical consideration. Therefore, the Black group was set as the reference category in all models and compared with the White nonreference group.

## Results

### Demographic Characteristics

A total of 8714 participants with TBI (mean [SD] age at index TBI, 49.0 [17.9] years; 3839 men [44.5%], 4700 women [54.5%], and 84 other gender [1.0%]; 2192 identifying as Black [25.2%] and 6522 as White [74.8%] race; 197 identifying as Hispanic or Latino [2.3%] and 8517 of non–Hispanic or Latino [97.7%] ethnicity) (Table 1). White participants were older at index TBI (mean [SD], 50.2 [18.6] years) compared with Black participants (mean [SD], 45.2 [15.2] years) (*P* < .001). The mean (SD) SDI of the cohort was 31.2 (6.2), with a lower SDI observed in White participants (mean [SD], 30.1 [5.3]) compared with Black participants (mean [SD], 34.6 [6.7]) (*P* < .001). The majority of TBI diagnoses were considered less serious (5935 participants [68.1%]), with no significant difference observed across racial groups.

**Table 1.** Cohort Characteristics

Variable	Participants, No. (%)				*P* value^a^
	All of Us, overall (N = 212 942)	Patients with TBI in All of Us			
		Overall (n = 8714)	Black (n = 2192)	White (n = 6522)	
Race					
Black	58 264 (27.4)	2192 (25.2)	NA	NA	NA
White	154 678 (72.6)	6522 (74.8)	NA	NA	
Ethnicity	212 942	8714	2192	6522	NA
Hispanic or Latino	5363 (2.5)	197 (2.3)	57 (2.6)	140 (2.1)	.25
Non–Hispanic or Latino	207 579 (97.5)	8517 (97.7)	2135 (97.4)	6382 (97.9)	
Gender^b^	210 561	8623	2157	6466	NA
Man	83 988 (39.9)	3839 (44.5)	1146 (53.1)	2693 (41.6)	<.001
Woman	125 612 (59.7)	4700 (54.5)	999 (46.3)	3701 (57.2)	
Other^c^	961 (0.5)	84 (1.0)	<20^d^	72 (1.1)	
Age at index TBI, mean (SD), y	NA	49.0 (17.9)	45.2 (15.2)	50.2 (18.6)	<.001
Deprivation measures, mean (SD)					
SDI (0-100 scale)^e^	32.1 (6.2)	31.2 (6.0)	34.6 (6.7)	30.1 (5.3)	<.001
Household income, $1000	64.7 (16.4)	64.6 (15.9)	59.7 (12.9)	66.2 (16.4)	<.001
Receive public assistance, %	14.5 (5.9)	14.4 (5.8)	18.2 (7.6)	13.1 (4.3)	<.001
Below poverty line, %	15.6 (5.3)	15.3 (5.3)	18.5 (6.0)	14.2 (4.5)	<.001
No health insurance, %	9.1 (4.2)	8.0 (3.9)	9.5 (4.0)	7.5 (3.7)	<.001
Attained a high school education, %	87.9 (5.3)	89.0 (5.0)	87.2 (5.3)	89.6 (4.7)	<.001
ICDPIC AIS					
Less serious (<3)	NA	5935 (68.1)	1471 (67.1)	4464 (68.4)	.26
Serious (≥3)	NA	2779 (31.9)	721 (32.9)	2058 (31.6)	

Abbreviations: ICDPIC AIS, International Classification of Diseases Program for Injury Categorization head or face Abbreviated Injury Score; NA, not applicable; SDI, social deprivation index; TBI, traumatic brain injury.

^a^ Comparison across racial groups performed using Pearson χ^2^ test and Welch 2-sample *t* test for categorical and continuous variables, respectively.

^b^ Missing data: overall, n = 2381; overall TBI, n = 91; Black or African American participants, n = 35; White participants, n = 56.

^c^ Included nonbinary; transgender; none of these describe me, and I would like to consider additional options; and prefer not to answer.

^d^ In accordance with the All of Us Data and Statistics Dissemination Policy, no group may be presented with participant or incident counts less than 20 (0 is acceptable).

^e^ Missing data: overall n = 119; overall TBI, n < 20; Black participants, n < 20; White participants, n < 20.

### Incident Neuropsychiatric Outcomes Following TBI

**Figure 1.**
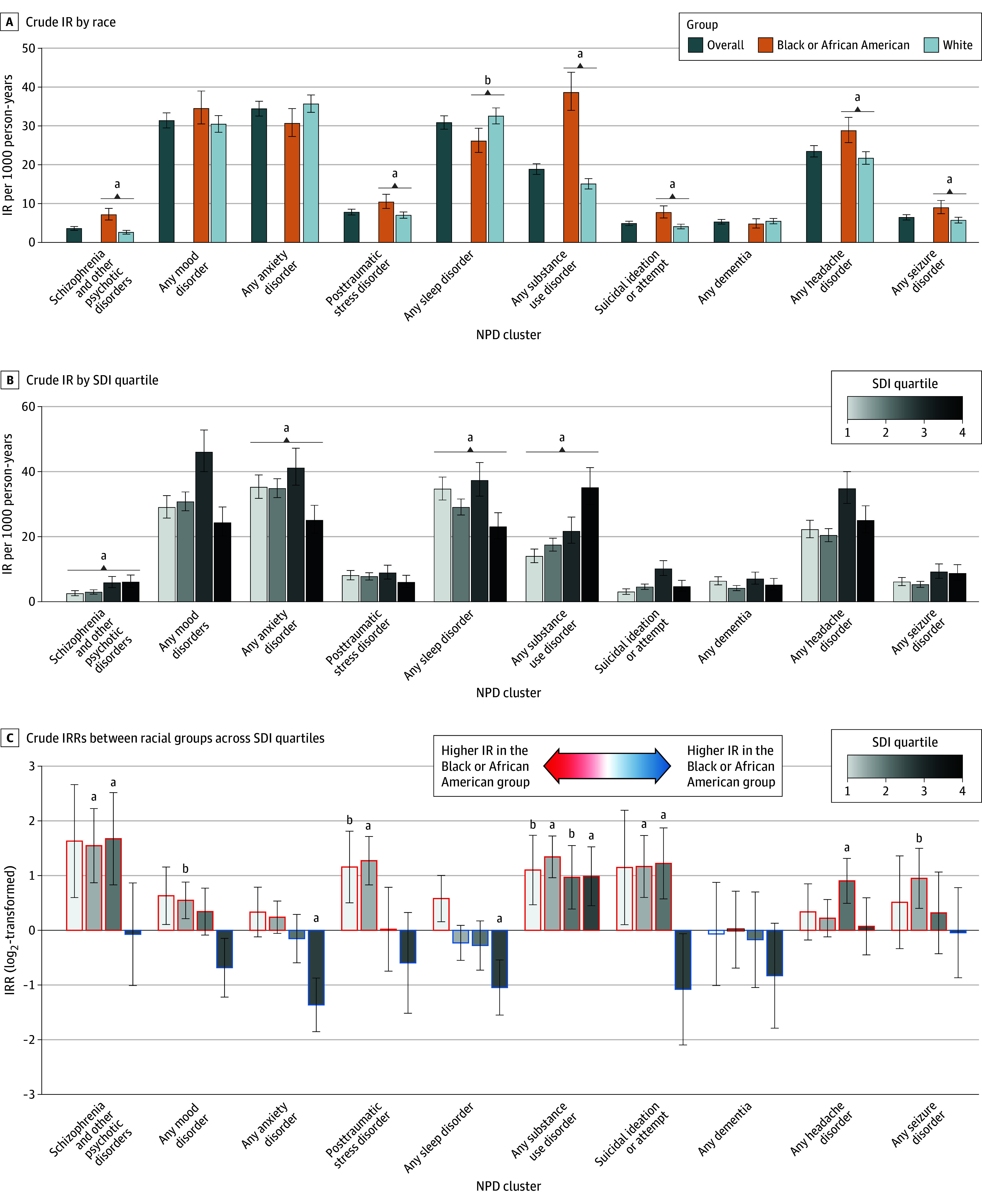
Crude Incidence Rates (IRs) Per 1000 Person-Years of Post–Traumatic Brain Injury Neuropsychiatric Diagnosis (NPD) Clusters A, Significance brackets show the difference between the Black group and the White group. B, Significance brackets show the difference between the lowest (quartile 1) and highest (quartile 4) social deprivation index (SDI) quartiles. Error bars indicate the 95% CIs. ^a^*P* < .001. ^b^*P* < .005.

Figure 1 displays the unadjusted (crude) IR for each NPD following TBI. Kaplan-Meier curves were generated to visualize NPDs across racial and SDI groups (eFigures 2 and 3 in Supplement 1). Across racial groups, the White group compared with the Black group had a significantly higher IR per 1000 person-years of post-TBI sleep disorders (32.48 [95% CI, 30.49-34.60] and 26.07 [95% CI, 23.15-29.35]; *P* = .001) and lower IRs per 1000 person-years of schizophrenia and other psychotic disorders (2.56 [95% CI, 2.13-3.08] and 7.09 [95% CI, 5.75-8.75]; *P* < .001), posttraumatic stress disorder (PTSD) (6.96 [95% CI, 6.20-7.82] and 10.39 [95% CI, 8.73-12.37]; *P* < .001), substance use disorders (SUDs) (15.02 [95% CI, 13.74-16.42] and 38.56 [95% CI, 33.97-43.77]; *P* < .001), suicidal ideation or suicide attempts (4.04 [95% CI, 3.48-4.68] and 7.68 [95% CI, 6.28-9.39]; *P* < .001), headache disorders (21.65 [95% CI, 20.09-23.32] and 28.74 [95% CI, 25.67-32.17]; *P* < .001), and seizure disorders (5.67 [95% CI, 4.98-6.46] and 8.92 [95% CI, 7.37-10.79]; *P* < .001) (Figure 1A; eTables 4 and 5 in Supplement 1). Stratified by SDI quartile, a significant difference in IR across SDI quartiles was observed for all NPD clusters except PTSD, which may have been driven by higher IR in SDI quartile 3 (Figure 1B; eTables 6 and 7 in Supplement 1). Compared with participants in the lowest deprivation quartile (quartile 1), those in the highest deprivation quartile (quartile 4) had a higher IR per 1000 person-years of schizophrenia and other psychotic disorders (6.01 [95% CI, 4.41-8.20] vs 2.48 [95% CI, 1.82-3.38]; *P* < .001) and SUDs (35.08 [95% CI, 29.83-41.26] vs 13.95 [95% CI, 12.00-16.21]; *P* < .001) and a lower IR per 1000 person-years of anxiety disorder (25.03 [95% CI, 21.12-29.66] vs 35.22 [95% CI, 31.81-39.00]; *P* < .001) and sleep disorder (23.03 [95% CI, 19.37-27.39] vs 34.65 [95% CI, 31.29-38.36]; *P* < .001).

Crude incidence rate ratios (IRRs) between the Black group and White group at each SDI quartile were then assessed (Figure 1C; eTable 8 in Supplement 1). At the low to middle deprivation level (quartile 2) in the Black group compared with the White group, higher rates of schizophrenia and other psychotic disorders (IRR, 2.92 [95% CI, 1.83-4.67]; *P* < .001), mood disorders (IRR, 1.46 [95% CI, 1.16-1.84]; *P* = .002), PTSD (IRR, 2.41 [95% CI, 1.78-3.28]; *P* < .001), suicidal ideation or suicide attempts (IRR, 2.25 [95% CI, 1.52-3.32]; *P* < .001), and seizure disorders (IRR, 1.93 [95% CI, 1.32-2.83]; *P* = .001) were observed. However, no association was found at higher deprivation levels (eTable 8 in Supplement 1). Conversely, at the highest level of deprivation (quartile 4) in the Black group compared with the White group, lower rates of anxiety disorders (IRR, 0.39 [95% CI, 0.28-0.55]; *P* < .001) and sleep disorders (IRR, 0.48 [95% CI, 0.34-0.69]; *P* < .001) were observed. The IRRs for post-TBI SUDs were higher in the Black group across all SDI quartiles (from 1.96 [95% CI, 1.31-2.93; *P* = .002] in quartile 3 to 2.54 [95% CI, 1.95-3.30; *P* < .001] in quartile 2).

### Association Among Race, SDI, and Post-TBI NPDs

**Figure 2.**
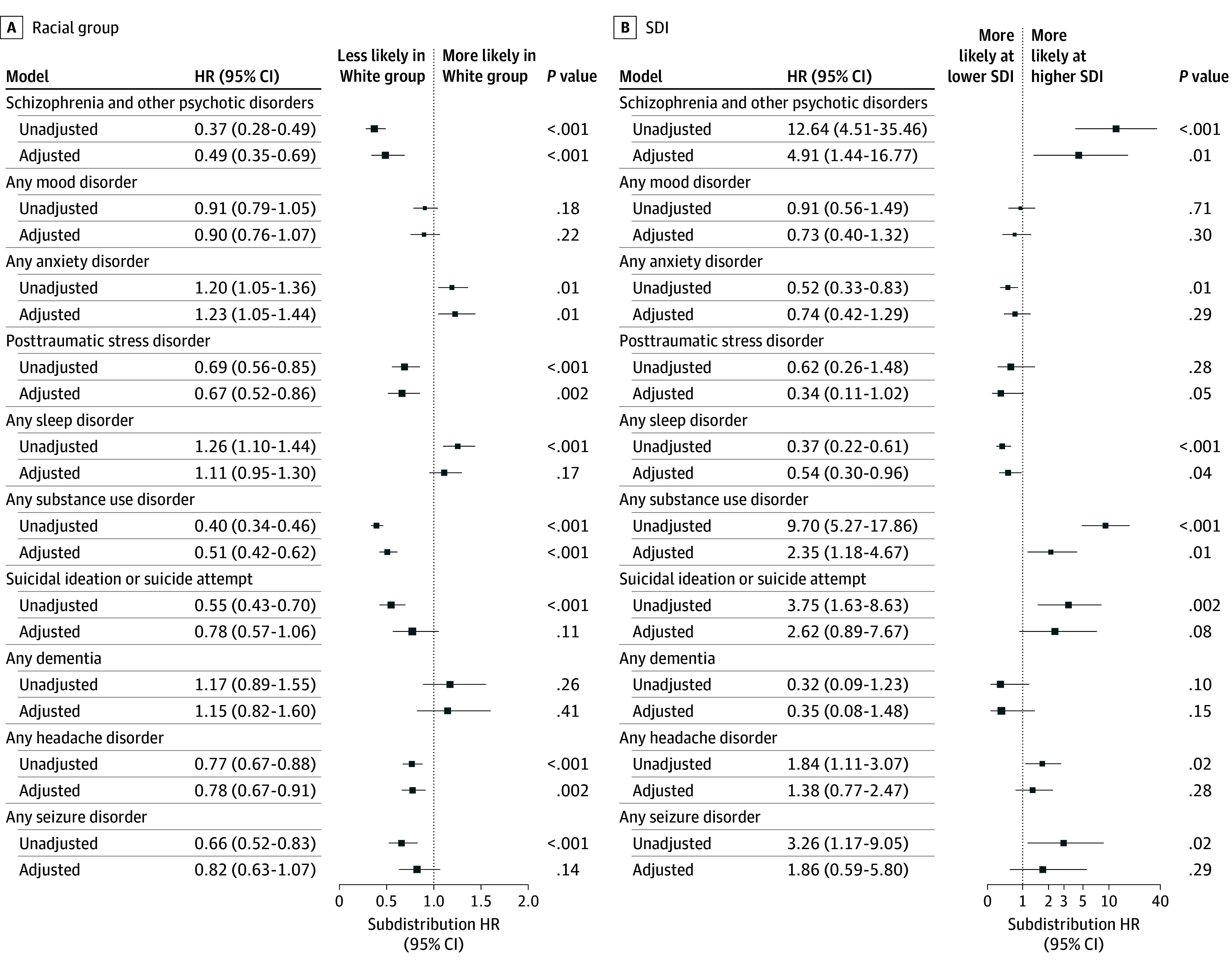
Competing-Risk Regression Coefficients Univariable and multivariable regression coefficients for each neuropsychiatric diagnostic cluster displayed as the subdistribution hazard ratio (HR) with 95% CI. The size of each box represents the relative size of each analytic sample per neuropsychiatric diagnostic model. SDI indicates social deprivation index.

Figure 2 displays the crude and adjusted subdistribution HRs across racial groups and SDI. The White group showed a higher risk of anxiety disorders in both the crude model (HR, 1.20 [95% CI, 1.05-1.36]) and adjusted model (adjusted HR [AHR], 1.23 [95% CI, 1.05-1.44]); however, no association was found after correction for multiple comparisons (Figure 2A). In the unadjusted model, increased risk of sleep disorder diagnosis was observed for the White group (HR, 1.26 [95% CI, 1.10-1.44]), although no association was found after model adjustment (AHR, 1.11 [95% CI, 0.95-1.30]). Compared with the Black group, the White group had a lower risk of schizophrenia and other psychotic disorders (AHR, 0.49 [95% CI, 0.35-0.69]), PTSD (AHR, 0.67 [95% CI, 0.52-0.86]), SUDs (AHR, 0.51 [95% CI, 0.42-0.62]), and headache disorders (AHR, 0.78 [95% CI, 0.67-0.91]). Additionally, the White group had a lower unadjusted risk of suicidal ideation or suicide attempts (HR, 0.55 [95% CI, 0.43-0.70]) and seizure disorder (HR, 0.66 [95% CI, 0.52-0.83]); however, no association was found after model adjustment.

The SDI did not provide a significant estimation of any NPD in adjusted models after correction for multiple testing (Figure 2B). In univariable analysis, higher SDI was associated with an increased risk of schizophrenia and other psychotic disorders (HR, 12.64 [95% CI, 4.51-35.46]) and SUDs (HR, 9.70 [95% CI, 5.27-17.86]). Unadjusted risks for suicidal ideation or suicide attempts (HR, 3.75 [95% CI, 1.63-8.63]), headache disorders (HR, 1.84 [95% CI, 1.11-3.07]), and seizure disorders (HR, 3.26 [95% CI, 1.17-9.05]) were associated with higher SDI, while lower SDI was associated with risk of anxiety disorders (HR, 0.52 [95% CI, 0.33-0.83]), though no association was found after multiple testing correction. An increased risk of sleep disorders was associated with lower SDI in both crude (HR, 0.37 [95% CI, 0.22-0.61]) and adjusted models (AHR, 0.54 [95% CI, 0.30-0.96]); however, no association was found after correction for multiple comparisons.

### Estimations After Correcting for Sample Selection Bias

The presence of nonrandom sample selection (ie, unobserved confounding) was assessed using the Lagrange multiplier test (Table 2). The null hypothesis of cohort exogeneity and random selection was rejected for all NPDs (*P* < .001) except schizophrenia and other psychotic disorders (*P* = .05). Next, we fit a series of bivariate probit models with sample selection, with coefficients representing the change in *z* score rather than the change in HR for a given outcome (Table 2). With all other covariables set to reference, the White group had a lower probability of being diagnosed with schizophrenia and other psychotic disorders (β [SE], −0.24 [0.07]; *P* < .001) and headache disorders (β [SE], −0.10 [0.03]; *P* = .003) compared with the Black group (eFigure 4 in Supplement 1). The White group showed a lower, albeit nonsignificant, probability of PTSD (β [SE], −0.13 [0.05]; *P* = .007) and SUDs (β [SE], −0.10 [0.04]; *P* = .006). The associations between racial group and all other NPDs were not statistically significant in the bivariate probit models.

**Table 2.** Sample Selection Model Coefficients

Diagnostic cluster	LM test *P* value^a^	Racial group^b^			SDI^b^		
		β (SE)	*P* value	*z* Score	β (SE)	*P* value	*z* Score
Schizophrenia and other psychotic disorders	.05	−0.24 (0.07)	<.001	−3.45	0.17 (0.29)	.55	0.59
Any mood disorder	<.001	0.01 (0.03)	.82	0.23	−0.65 (0.14)	<.001	−4.70
Any anxiety disorder	<.001	0.05 (0.03)	.11	1.60	−0.85 (0.13)	<.001	−6.59
PTSD	<.001	−0.13 (0.05)	.007	−2.72	−1.06 (0.22)	<.001	−4.82
Any sleep disorder	<.001	0.02 (0.02)	.21	1.24	0.34 (0.08)	<.001	4.39
Any SUD	<.001	−0.10 (0.04)	.006	−2.73	−0.14 (0.15)	.35	−0.93
Suicidal ideation or suicide attempt	<.001	−0.09 (0.05)	.09	−1.71	−0.23 (0.25)	.34	−0.95
Any dementia	<.001	0.05 (0.06)	.43	0.78	−1.03 (0.22)	<.001	−4.57
Any headache disorder	<.001	−0.10 (0.03)	.003	−3.02	−0.52 (0.14)	<.001	−3.75
Any seizure disorder	<.001	−0.08 (0.05)	.12	−1.54	−0.46 (0.21)	.03	−2.17

Abbreviations: LM, Lagrange multiplier; PTSD, posttraumatic stress disorder; SDI, social deprivation index; SUD, substance use disorder.

^a^ The LM test was first performed for each model to assess nonrandom sample selection.

^b^ Coefficients for racial group (Black group compared with White group) and SDI are displayed for each adjusted bivariate probit model with sample selection; coefficients represent those of the outcome equation. Significance after correction for multiple testing was determined as *P* < .005.

Considering the contribution of SDI, low levels of deprivation were observed to have a higher estimated probability for mood disorders (β [SE], −0.65 [0.14]; *P* < .001); anxiety disorders (β [SE], −0.85 [0.13]; *P* < .001); PTSD (β [SE], −1.06 [0.22]; *P* < .001); any dementia (β [SE], −1.03 [0.22]; *P* < .001); and headache disorders (β [SE], −0.52 [0.14]; *P* < .001) compared with the highest SDI (eFigure 4 in Supplement 1). An increased probability of seizure disorders, albeit nonsignificant, was observed at a lower SDI (β [SE], −0.46 [0.21]; *P* = .03). Conversely, higher levels of deprivation were associated with a higher probability of sleep disorders (β [SE], 0.34 [0.08]; *P* < .001) compared with low SDI, indicating a change in the direction of association compared with competing-risk models.

## Discussion

This cohort study leveraged the All of Us resource to explore the associations between BPSE prism factors and post-TBI trajectory.^4^ The findings highlight both the significant association between post-TBI NPD and race and how unmeasured factors may contribute to differences in the incidence of post-TBI NPDs among Black participants and White participants with relative social advantage or disadvantage. In competing-risk models, White participants had a decreased risk of diagnosis in 4 of the 10 NPD clusters assessed compared with Black participants. Accounting for sample selection showed that White participants had a lower probability of schizophrenia and other psychotic disorders and headache disorders compared with Black participants. Interestingly, SDI was not a significant estimator in any adjusted competing-risk model, although univariable analysis showed an increased risk of schizophrenia and psychotic disorders and SUDs at higher deprivation levels. In bivariate probit models, we observed a higher probability of diagnosis at a lower SDI in 6 of the 10 NPD clusters assessed. Together, these results suggest the importance of considering the influence of sample selection and that a complex interplay among race, social deprivation, and sample selection may be present.

Following neurotrauma, some groups or individuals may be less likely to receive a diagnosis of TBI. Unequal access to health care may prevent diagnosis of trauma,^6,7^ and once in a health care setting, diagnosis of TBI may differ across ethnoracial groups.^41^ Additionally, individuals diagnosed with psychotic disorders, mood disorders, anxiety disorders, and conduct disorder may confer a higher risk of sustaining a TBI,^42,43,44^ suggesting that the prevalence of preexisting NPDs may differ among populations with and without TBI. We found evidence of selection bias for all NPDs except schizophrenia and other psychotic disorders, suggesting that schizophrenia or other psychoses may be less influenced by selection bias as it relates to TBI diagnosis and participation in the All of Us Research Program.

The significant association between racial group and diagnosis of schizophrenia and other psychotic disorders and headache disorders suggests that other unmeasured pervasive societal structures are still not accounted for, although adjustment for other demographic factors and selection bias attenuated the observed associations. We propose that the differences observed between the Black group and White group were not due to race itself but, rather, serves as a surrogate for the effects of structural racism.^6,7^ Associations between the Black group and a higher probability of NPDs were maintained after model adjustment and accounting for sample selection, suggesting disparate outcomes associated with sample selection across racial groups. These findings corroborate previous research investigating racial disparities in post-TBI neuropsychiatric outcomes.^45^ Greater social deprivation was associated with a lower risk for many of the NPD clusters in bivariate probit models, suggesting that social deprivation based on post-TBI NPD may be connected to a person’s ability to access and use health care settings in which many NPDs are diagnosed, as well as participate in research studies.^45,46,47^

### Limitations

This study had some limitations. First, the study involved the use of database-derived data and their incorporated methodology. The study relied on the All of Us database, which may contain inherent biases associated with diagnostic billing codes, missing information, and participation. Second, these findings may not be generalizable to other populations due to demographic differences, and future studies should expand analyses to other racial groups and geographic areas. Efforts to improve data collection methods and address potential biases are necessary to ameliorate such limitations. Finally, differences in health care systems, cultural contexts, and population characteristics may influence the applicability of the results; therefore, replicating the study in more diverse populations or using more diverse databases may enhance external validity.

## Conclusions

This cohort study of All of Us Research Program participants with TBI identified significant racial disparities in neuropsychiatric outcomes following TBI, with White participants observed to have a lower risk of being diagnosed with a psychotic disorder, PTSD, SUD, or headache disorder compared with Black participants. The application of Heckman-type selection models indicated that disparities may be influenced by the interplay between unobserved social and structural factors that unequally impact selection bias. Together, the findings of this study corroborate the National Academies of Sciences, Engineering, and Medicine’s call for framing TBI as a chronic condition requiring ongoing care rather than as an isolated event.^4^ This approach highlights the substantial care gap and lack of support services for patients after TBI. This study underscores the importance of addressing both psychosocial and environmental modifiers that may alter the trajectory of patients who sustain TBI.^48^ Future research should focus on identifying specific social and structural factors contributing to these disparities and further integrate BPSE prism factors associated with TBI outcomes.

## Notes

Supplement 1. **eTable 1.** Number of Participants for Each Neuropsychiatric Disorder (NPD) Analytic Sample **eTable 2.** Univariable Competing-Risk Regression Coefficients for Each Preexisting Neuropsychiatric Disorder **eTable 3.** Demographic and Clinical Characteristics of the Control Cohort Used in the Heckman-Type Selection Model for Selection Into the TBI Cohort **eTable 4.** Crude Incidence Rates (IRs) of NPD Diagnostic Clusters (Condition) in Full Cohort (Full) and in Black or African American and White Racial Groups **eTable 5.** Comparison of Post-TBI NPD (Condition) Crude Incidence Rates Across Racial Groups **eTable 6.** Crude Incidence Rates (IRs) of NPD Diagnostic Clusters (Condition) Across Social Deprivation Index (SDI) Quartiles **eTable 7.** Comparison of Post-TBI NPD Crude Incidence Rates Across SDI Quartiles **eTable 8.** Comparison of Post-TBI NPD Crude Incidence Rates Between Racial Groups Across Social Deprivation Index (SDI) Quartiles **eFigure 1.** The Proportion of Participants in the TBI Cohort (n = 8714) Included for Each Neuropsychiatric Disorder (NPD) Analysis **eFigure 2.** Kaplan-Meier Curves Stratified by Racial Group **eFigure 3.** Kaplan-Meier Curves Stratified by Social Deprivation Index (SDI) Quartile **eFigure 4.** Estimated Probabilities From Bivariate Probit Models (BPM)
eMethods.

eReferences.

Supplement 2.
Data Sharing Statement
