# Supplementary material for: Inequities in Neuropsychiatric Outcomes After Brain Trauma in the All of Us Database
Source: JAMA Netw Open. 2025 Oct 24;8(10):e2539313. doi: 10.1001/jamanetworkopen.2025.39313 (PMC12552927; doi:10.1001/jamanetworkopen.2025.39313)
Supplement: Supplement 1. — eTable 1. Number of Participants for Each Neuropsychiatric Disorder (NPD) Analytic Sample eTable 2. Univariable Competing-Risk Regression Coefficients for Each Preexisting Neuropsychiatric Disorder eTable 3. Demographic and Clinical Characteristics of the Control Cohort Used in the Heckman-Type Selection Model for Selection Into the TBI Cohort eTable 4. Crude Incidence Rates (IRs) of NPD Diagnostic Clusters (Condition) in Full Cohort (Full) and in Black or African American and White Racial Groups eTable 5. Comparison of Post-TBI NPD (Condition) Crude Incidence Rates Across Racial Groups eTable 6. Crude Incidence Rates (IRs) of NPD Diagnostic Clusters (Condition) Across Social Deprivation Index (SDI) Quartiles eTable 7. Comparison of Post-TBI NPD Crude Incidence Rates Across SDI Quartiles eTable 8. Comparison of Post-TBI NPD Crude Incidence Rates Between Racial Groups Across Social Deprivation Index (SDI) Quartiles eFigure 1. The Proportion of Participants in the TBI Cohort (n = 8714) Included for Each Neuropsychiatric Disorder (NPD) Analysis eFigure 2. Kaplan-Meier Curves Stratified by Racial Group eFigure 3. Kaplan-Meier Curves Stratified by Social Deprivation Index (SDI) Quartile eFigure 4. Estimated Probabilities From Bivariate Probit Models (BPM) eMethods. eReferences. [file jamanetwopen-e2539313-s001.pdf]

Supplemental Online Content

Wroblewski TH, Ononogbu-Uche FC, Jagiani P, et al. Neuropsychiatic outcomes after brain trauma in the All of Us database. *JAMA Netw Open*. 2025;8(10):e2539313. doi:10.1001/jamanetworkopen.2025.39313

eTable 1. Number of Participants for Each Neuropsychiatric Disorder (NPD) Analytic Sample

eTable 2. Univariable Competing-Risk Regression Coefficients for Each Preexisting Neuropsychiatric Disorder

eTable 3. Demographic and Clinical Characteristics of the Control Cohort Used in the Heckman-Type Selection Model for Selection Into the TBI Cohort

eTable 4. Crude Incidence Rates (IRs) of NPD Diagnostic Clusters (Condition) in Full Cohort (Full) and in Black or African American and White Racial Groups

eTable 5. Comparison of Post-TBI NPD (Condition) Crude Incidence Rates Across Racial Groups

eTable 6. Crude Incidence Rates (IRs) of NPD Diagnostic Clusters (Condition) Across Social Deprivation Index (SDI) Quartiles

eTable 7. Comparison of Post-TBI NPD Crude Incidence Rates Across SDI Quartiles

eTable 8. Comparison of Post-TBI NPD Crude Incidence Rates Between Racial Groups Across Social Deprivation Index (SDI) Quartiles

eFigure 1. The Proportion of Participants in the TBI Cohort (n = 8714) Included for Each Neuropsychiatric Disorder (NPD) Analysis

eFigure 2. Kaplan-Meier Curves Stratified by Racial Group

eFigure 3. Kaplan-Meier Curves Stratified by Social Deprivation Index (SDI) Quartile

eFigure 4. Estimated Probabilities From Bivariate Probit Models (BPM)

eMethods.

eReferences.

This supplemental material has been provided by the authors to give readers additional information about their work.

© 2025 Wroblewski TH et al. *JAMA Network Open*.

eTable 1. Number of Participants for Each Neuropsychiatric Disorder (NPD) Analytic Sample. Participants are stratified by total number at risk, number censored at the end of the EHR span, number who were diagnosed with the incident NPD, or number who were deceased prior to incident diagnosis of NPD of interest.

Diagnostic Cluster	Total at Risk (n)	Censored, No. (%)	Incident NPD diagnosis, No. (%)	Deceased, No. (%)
Schizophrenia and other psychotic disorders	7,287	6,934 (95.2)	199 (2.7)	154 (2.1)
Any mood disorder	4,546	3,463 (76.2)	1,016 (22.3)	67 (1.5)
Any anxiety disorder	5,048	3,701 (73.3)	1,261 (25.0)	86 (1.7)
PTSD	7,025	6,461 (92.0)	412 (5.9)	152 (2.2)
Any sleep disorder	5,479	4,165 (76.0)	1,233 (22.5)	81 (1.5)
Any SUD	5,199	4,391 (84.5)	725 (13.9)	83 (1.6)
Suicidal ideation or attempt	7,219	6,790 (94.1)	270 (3.7)	159 (2.2)
Dementias	7,337	6,911 (94.2)	295 (4.0)	131 (1.8)
Any headache disorder	5,645	4,537 (80.4)	994 (17.6)	114 (2.0)
Any seizure disorder	6,798	6,332 (93.1)	335 (4.9)	131 (1.9)

© 2025 Wroblewski TH et al. *JAMA Network Open*.

eTable 2. Univariable Competing-Risk Regression Coefficients for Each Preexisting Neuropsychiatric Disorder

Outcome	Group	HR [95%CI]	P Value
Schizophrenia and other psychotic disorders (SCZ)	Pre-existing MOOD	2.31 [1.74-3.07]	<.001
	Pre-existing ANX	1.69 [1.25-2.28]	<.001
	Pre-existing PTSD	1.78 [1.08-2.93]	0.02
	Pre-existing SLEEP	0.92 [0.64-1.34]	0.67
	Pre-existing SUD	2.46 [1.85-3.28]	<.001
	Pre-existing SUIC	5.45 [3.55-8.36]	<.001
	Pre-existing DEM	1.02 [0.38-2.75]	0.97
	Pre-existing HACHE	0.92 [0.62-1.36]	0.67
	Pre-existing SEIZ	1.51 [0.94-2.42]	0.09
Any mood disorder (MOOD)	Pre-existing SCZ	2.06 [1.22-3.49]	0.007
	Pre-existing ANX	2.23 [1.85-2.70]	<.001
	Pre-existing PTSD	2.58 [1.60-4.16]	<.001
	Pre-existing SLEEP	0.93 [0.75-1.15]	0.49
	Pre-existing SUD	1.56 [1.31-1.84]	<.001
	Pre-existing SUIC	1.62 [0.69-3.84]	0.27
	Pre-existing DEM	0.75 [0.31-1.79]	0.51
	Pre-existing HACHE	1.41 [1.16-1.71]	<.001
	Pre-existing SEIZ	1.21 [0.90-1.62]	0.2
Any anxiety disorder (ANX)	Pre-existing SCZ	1.57 [1.08-2.29]	0.02
	Pre-existing MOOD	2.33 [2.05-2.66]	<.001
	Pre-existing PTSD	NA	NA
	Pre-existing SLEEP	1.22 [1.04-1.44]	0.01
	Pre-existing SUD	1.52 [1.32-1.75]	<.001
	Pre-existing SUIC	2.54 [1.78-3.62]	<.001
	Pre-existing DEM	1.46 [0.92-2.32]	0.11
	Pre-existing HACHE	1.78 [1.52-2.10]	<.001
	Pre-existing SEIZ	1.08 [0.83-1.40]	0.59
Post-traumatic stress disorder (PTSD)	Pre-existing SCZ	1.96 [1.24-3.11]	0.004
	Pre-existing MOOD	2.65 [2.18-3.22]	<.001
	Pre-existing ANX	2.29 [1.86-2.82]	<.001
	Pre-existing SLEEP	1.22 [0.96-1.56]	0.1
	Pre-existing SUD	2.30 [1.88-2.82]	<.001
	Pre-existing SUIC	2.90 [1.93-4.36]	<.001
	Pre-existing DEM	1.24 [0.63-2.43]	0.53
	Pre-existing HACHE	2.07 [1.65-2.59]	<.001
	Pre-existing SEIZ	1.72 [1.25-2.37]	<.001
Any sleep disorder (SLEEP)	Pre-existing SCZ	1.24 [0.90-1.71]	0.18
	Pre-existing MOOD	1.86 [1.64-2.10]	<.001
	Pre-existing ANX	1.87 [1.64-2.14]	<.001
	Pre-existing PTSD	1.79 [1.40-2.30]	<.001
	Pre-existing SUD	1.23 [1.08-1.41]	0.002
	Pre-existing SUIC	1.47 [1.08-1.99]	0.01
	Pre-existing DEM	1.47 [0.97-2.23]	0.07

© 2025 Wroblewski TH et al. *JAMA Network Open*.

	Pre-existing HACHE	1.92 [1.64-2.24]	<.001
	Pre-existing SEIZ	1.27 [1.01-1.59]	0.04
Substance use disorders (SUD)	Pre-existing SCZ	1.28 [0.69-2.39]	0.44
	Pre-existing MOOD	1.10 [0.93-1.31]	0.28
	Pre-existing ANX	1.10 [0.91-1.33]	0.32
	Pre-existing PTSD	1.60 [1.12-2.28]	0.009
	Pre-existing SLEEP	0.74 [0.60-0.91]	0.005
	Pre-existing SUIC	1.03 [0.47-2.29]	0.94
	Pre-existing DEM	1.06 [0.60-1.85]	0.85
	Pre-existing HACHE	0.75 [0.60-0.95]	0.02
	Pre-existing SEIZ	0.98 [0.69-1.38]	0.89
Suicidal ideation or attempt (SUIC)	Pre-existing SCZ	4.19 [2.74-6.39]	<.001
	Pre-existing MOOD	2.42 [1.90-3.09]	<.001
	Pre-existing ANX	2.03 [1.58-2.62]	<.001
	Pre-existing PTSD	2.54 [1.72-3.73]	<.001
	Pre-existing SLEEP	0.94 [0.69-1.29]	0.72
	Pre-existing SUD	3.45 [2.71-4.38]	<.001
	Pre-existing DEM	0.71 [0.26-1.90]	0.49
	Pre-existing HACHE	1.11 [0.81-1.53]	0.52
	Pre-existing SEIZ	2.05 [1.42-2.95]	<.001
Dementias (DEM)	Pre-existing SCZ	3.16 [2.12-4.72]	<.001
	Pre-existing MOOD	2.62 [2.07-3.31]	<.001
	Pre-existing ANX	2.06 [1.62-2.63]	<.001
	Pre-existing PTSD	2.31 [1.59-3.33]	<.001
	Pre-existing SLEEP	1.78 [1.37-2.31]	<.001
	Pre-existing SUD	2.13 [1.68-2.71]	<.001
	Pre-existing SUIC	3.04 [2.02-4.56]	<.001
	Pre-existing HACHE	1.08 [0.79-1.48]	0.62
	Pre-existing SEIZ	2.34 [1.68-3.27]	<.001
Any headache disorder (HACHE)	Pre-existing SCZ	1.33 [0.97-1.82]	0.08
	Pre-existing MOOD	1.41 [1.24-1.62]	<.001
	Pre-existing ANX	1.43 [1.23-1.66]	<.001
	Pre-existing PTSD	1.31 [0.99-1.73]	0.06
	Pre-existing SLEEP	1.27 [1.07-1.50]	0.005
	Pre-existing SUD	1.23 [1.06-1.42]	0.006
	Pre-existing SUIC	1.80 [1.35-2.40]	<.001
	Pre-existing DEM	1.55 [1.09-2.21]	0.02
	Pre-existing SEIZ	1.21 [0.94-1.56]	0.14
Any seizure disorder (SEIZ)	Pre-existing SCZ	1.96 [1.23-3.15]	0.005
	Pre-existing MOOD	1.16 [0.92-1.45]	0.22
	Pre-existing ANX	1.17 [0.91-1.50]	0.21
	Pre-existing PTSD	1.56 [1.05-2.34]	0.03
	Pre-existing SLEEP	0.87 [0.64-1.16]	0.33
	Pre-existing SUD	1.89 [1.51-2.38]	<.001
	Pre-existing SUIC	2.16 [1.37-3.40]	<.001
	Pre-existing DEM	1.99 [1.12-3.52]	0.02
	Pre-existing HACHE	1.01 [0.75-1.38]	0.94

© 2025 Wroblewski TH et al. *JAMA Network Open*.

eTable 3. Demographic and Clinical Characteristics of the Control Cohort Used in the Heckman-Type Selection Model for Selection Into the TBI Cohort

Variable	N	Overall, No. (%)	Black or African American, No. (%)	White, No. (%)
Overall (N)		39,393	7,393	21,839
Ethnicity	38,352			
Not Hispanic or Latino		30,483 (79)	7,243 (98)	21,274 (97)
Hispanic or Latino		7,503 (20)	150 (2.0)	565 (2.6)
Other		366 (1.0)	0 (0)	0 (0)
Not Reported		1,041	0	0
Gender	38,602			
Man		14,405 (37)	2,778 (38)	8,478 (39)
Woman		24,018 (62)	4,478 (61)	13,079 (60)
Other		179 (0.5)	33 (0.5)	110 (0.5)
Not Reported		791	104	172
Deprivation measures, mean ± SD	39,355			
Social Deprivation Index (0-100)		32.4±6.4	35.6±5.8	30.5±5.8
Median household income ($X1000)		65.6±16.8	59.7±12.8	67.3±17.6
Receive public assistance (%)		14.9±6.4	18.3±6.5	13.1±4.9
Below poverty line (%)		15.7±5.2	18.5±5.0	14.2±4.8
No health insurance (%)		9.5±4.4	11.2±4.1	8.4±4.1
Attained high school education (%)		87.1±5.8	85.7±5.0	88.8±5.0
Not Reported		38	<20	<20
NPD Diagnostic Clusters	39,393			
Schizophrenia & other psychotic disorders		785 (2.0)	339 (4.6)	273 (1.3)
Any mood disorder		8,735 (22)	1,600 (22)	5,182 (24)
Any anxiety disorder		8,464 (21)	1,233 (17)	5,391 (25)
PTSD		1,266 (3.2)	282 (3.8)	686 (3.1)
Any sleep disorder		8,556 (22)	1,408 (19)	5,483 (25)
Any SUD		6,199 (16)	1,902 (26)	2,975 (14)
Suicidal ideation or attempt		698 (1.8)	202 (2.7)	321 (1.5)
Dementias		454 (1.2)	86 (1.2)	263 (1.2)
Any headache disorder		5,363 (14)	873 (12)	3,025 (14)
Any seizure disorder		1,050 (2.7)	226 (3.1)	564 (2.6)

© 2025 Wroblewski TH et al. *JAMA Network Open*.

eTable 4. Crude Incidence Rates (IRs) of NPD Diagnostic Clusters (Condition) in Full Cohort (Full) and in Black or African American and White Racial Groups. Rates reported in 1,000 person-years and 95% confidence intervals.

Group	Condition	IR [95% CI]	n Events	n Total
Full	SCZ	3.55 [3.09-4.08]	199	7287
	ANX	34.36 [32.52-36.31]	1261	5048
	MOOD	31.33 [29.46-33.31]	1016	4546
	SLEEP	30.80 [29.13-32.57]	1233	5479
	HACHE	23.40 [21.99-24.90]	994	5645
	SUD	18.80 [17.48-20.22]	725	5199
	PTSD	7.74 [7.03-8.53]	412	7025
	SEIZ	6.41 [5.76-7.13]	335	6798
	DEM	5.27 [4.70-5.90]	295	7337
	SUIC	4.85 [4.30-5.46]	270	7219
Black or African American	SCZ	7.09 [5.75-8.75]	87	1673
	MOOD	34.45 [30.47-38.95]	255	1111
	ANX	30.63 [27.26-34.41]	283	1331
	PTSD	10.39 [8.73-12.37]	126	1694
	SLEEP	26.07 [23.15-29.35]	273	1488
	SUD	38.56 [33.97-43.77]	239	915
	SUIC	7.68 [6.28-9.39]	95	1679
	DEM	4.74 [3.71-6.07]	63	1793
	HACHE	28.74 [25.67-32.17]	302	1446
	SEIZ	8.92 [7.37-10.79]	106	1630
White	SCZ	2.56 [2.13-3.08]	112	5614
	MOOD	30.40 [28.32-32.64]	761	3435
	ANX	35.62 [33.46-37.92]	978	3717
	PTSD	6.96 [6.20-7.82]	286	5331
	SLEEP	32.48 [30.49-34.60]	960	3991
	SUD	15.02 [13.74-16.42]	486	4284
	SUIC	4.04 [3.48-4.68]	175	5540
	DEM	5.43 [4.77-6.17]	232	5544
	HACHE	21.65 [20.09-23.32]	692	4199
	SEIZ	5.67 [4.98-6.46]	229	5168

© 2025 Wroblewski TH et al. *JAMA Network Open*.

eTable 5. Comparison of Post-TBI NPD (Condition) Crude Incidence Rates Across Racial Groups. Significance determined by log rank test with significance level set as P<.005.

Condition	P value (log rank test)
SCZ	<.001
MOOD	0.08
ANX	0.02
PTSD	<.001
SLEEP	0.001
SUD	<.001
SUIC	<.001
DEM	0.34
HACHE	<.001
SEIZ	<.001

© 2025 Wroblewski TH et al. *JAMA Network Open*.

eTable 6. Crude Incidence Rates (IRs) of NPD Diagnostic Clusters (Condition) Across Social Deprivation Index (SDI) Quartiles. Rates reported in 1000 person-years +/- 95% confidence intervals, upper and lower.

Condition	SDI Quartile	IR [95% CI]	n Events	n Total
SCZ	1	2.48 [1.82-3.38]	40	2108
SCZ	2	2.91 [2.32-3.66]	74	2623
SCZ	3	5.79 [4.32-7.75]	45	1533
SCZ	4	6.01 [4.41-8.20]	40	1023
MOOD	1	28.97 [25.72-32.64]	270	1320
MOOD	2	30.75 [27.98-33.79]	432	1549
MOOD	3	46.01 [40.04-52.87]	199	946
MOOD	4	24.27 [20.22-29.14]	115	731
ANX	1	35.22 [31.81-39.00]	370	1449
ANX	2	34.81 [32.04-37.83]	556	1767
ANX	3	41.16 [35.86-47.24]	202	1019
ANX	4	25.03 [21.12-29.66]	133	813
PTSD	1	8.02 [6.71-9.59]	121	1990
PTSD	2	7.72 [6.68-8.91]	185	2507
PTSD	3	8.85 [6.96-11.24]	67	1520
PTSD	4	5.93 [4.33-8.11]	39	1008
SLEEP	1	34.65 [31.29-38.36]	370	1480
SLEEP	2	29.03 [26.67-31.60]	535	2040
SLEEP	3	37.30 [32.47-42.84]	200	1118
SLEEP	4	23.03 [19.37-27.39]	128	841
SUD	1	13.95 [12.00-16.21]	170	1618
SUD	2	17.43 [15.55-19.53]	296	1858
SUD	3	21.65 [18.01-26.03]	113	1028
SUD	4	35.08 [29.83-41.26]	146	695
SUIC	1	2.97 [2.24-3.95]	48	2078
SUIC	2	4.52 [3.76-5.43]	114	2606
SUIC	3	10.10 [8.08-12.63]	77	1515
SUIC	4	4.61 [3.24-6.55]	31	1020
DEM	1	6.30 [5.17-7.67]	99	2076
DEM	2	4.12 [3.40-4.98]	105	2641
DEM	3	7.00 [5.39-9.10]	56	1585
DEM	4	5.14 [3.69-7.16]	35	1035
HACHE	1	22.21 [19.67-25.07]	262	1595
HACHE	2	20.38 [18.47-22.49]	397	2052
HACHE	3	34.78 [30.23-40.03]	195	1142
HACHE	4	25.01 [21.19-29.52]	140	856
SEIZ	1	6.07 [4.94-7.46]	91	1968
SEIZ	2	5.24 [4.40-6.23]	126	2479
SEIZ	3	9.13 [7.17-11.62]	66	1423
SEIZ	4	8.68 [6.62-11.40]	52	928

© 2025 Wroblewski TH et al. *JAMA Network Open*.

eTable 7. Comparison of Post-TBI NPD Crude Incidence Rates Across SDI Quartiles. Significance level set as P<.005.

Condition	SDI Quartile	Group P value (log rank test)	P value (exact Poisson)
SCZ	1	<.001	NA
SCZ	2	<.001	0.41
SCZ	3	<.001	<.001
SCZ	4	<.001	<.001
MOOD	1	<.001	NA
MOOD	2	<.001	0.44
MOOD	3	<.001	<.001
MOOD	4	<.001	0.11
ANX	1	<.001	NA
ANX	2	<.001	0.86
ANX	3	<.001	0.075
ANX	4	<.001	<.001
PTSD	1	0.23	NA
PTSD	2	0.23	0.74
PTSD	3	0.23	0.52
PTSD	4	0.23	0.10
SLEEP	1	<.001	NA
SLEEP	2	<.001	0.009
SLEEP	3	<.001	0.40
SLEEP	4	<.001	<.001
SUD	1	<.001	NA
SUD	2	<.001	0.021
SUD	3	<.001	<.001
SUD	4	<.001	<.001
SUIC	1	<.001	NA
SUIC	2	<.001	0.015
SUIC	3	<.001	<.001
SUIC	4	<.001	0.058
DEM	1	0.003	NA
DEM	2	0.003	0.002
DEM	3	0.003	0.53
DEM	4	0.003	0.30
HACHE	1	<.001	NA
HACHE	2	<.001	0.28
HACHE	3	<.001	<.001
HACHE	4	<.001	0.26
SEIZ	1	<.001	NA
SEIZ	2	<.001	0.28
SEIZ	3	<.001	0.012
SEIZ	4	<.001	0.040

© 2025 Wroblewski TH et al. *JAMA Network Open*.

eTable 8. Comparison of Post-TBI NPD Crude Incidence Rates Between Racial Groups Across Social Deprivation Index (SDI) Quartiles. Crude incidence rate ratio (IRR) calculated by comparing crude incidence rate (IR) in Black or African American group versus IR in White group. P value represents log-rank test (chi-squared statistic). Significance level set as P<.005.

Condition	Group	SDI Quant	IR [95% CI]	n Events	n Total	IRR [95% CI]	P value (log rank test)
SCZ	Black or African American	1	6.37 [3.43-11.84]	<20	217	3.1 [1.51-6.33]	0.005
SCZ	White	1	2.06 [1.44-2.94]	30	1891	NA	
SCZ	Black or African American	2	6.39 [4.41-9.25]	28	520	2.92 [1.83-4.67]	<.001
SCZ	White	2	2.19 [1.64-2.92]	46	2103	NA	
SCZ	Black or African American	3	11.99 [7.97-18.04]	23	373	3.19 [1.78-5.73]	<.001
SCZ	White	3	3.76 [2.47-5.70]	22	1160	NA	
SCZ	Black or African American	4	5.91 [4.02-8.68]	26	563	0.95 [0.5-1.82]	0.88
SCZ	White	4	6.21 [3.68-10.49]	<20	460	NA	
MOOD	Black or African American	1	42.92 [30.51-60.38]	33	119	1.55 [1.08-2.23]	0.026
MOOD	White	1	27.72 [24.41-31.48]	237	1201	NA	
MOOD	Black or African American	2	41.99 [34.15-51.62]	90	291	1.46 [1.16-1.84]	0.002
MOOD	White	2	28.72 [25.83-31.93]	342	1258	NA	
MOOD	Black or African American	3	54.37 [42.56-69.46]	64	266	1.27 [0.94-1.71]	0.12
MOOD	White	3	42.88 [36.23-50.76]	135	680	NA	
MOOD	Black or African American	4	20.53 [16.19-26.04]	68	435	0.62 [0.43-0.9]	0.014
MOOD	White	4	32.96 [24.77-43.87]	47	296	NA	
ANX	Black or African American	1	43.30 [32.22-58.18]	44	145	1.26 [0.92-1.73]	0.16
ANX	White	1	34.36 [30.82-38.30]	326	1304	NA	
ANX	Black or African American	2	39.79 [33.14-47.77]	115	378	1.18 [0.96-1.45]	0.12
ANX	White	2	33.71 [30.71-37.01]	441	1389	NA	
ANX	Black or African American	3	38.21 [29.47-49.54]	57	307	0.9 [0.66-1.22]	0.50
ANX	White	3	42.44 [36.07-49.95]	145	712	NA	
ANX	Black or African American	4	17.44 [13.72-22.16]	67	501	0.39 [0.28-0.55]	<.001
ANX	White	4	44.84 [35.23-57.08]	66	312	NA	
PTSD	Black or African American	1	16.01 [10.64-24.10]	23	203	2.23 [1.42-3.51]	0.001
PTSD	White	1	7.18 [5.89-8.76]	98	1787	NA	
PTSD	Black or African American	2	15.04 [11.70-19.32]	61	504	2.41 [1.78-3.28]	<.001
PTSD	White	2	6.23 [5.22-7.42]	124	2003	NA	

© 2025 Wroblewski TH et al. *JAMA Network Open*.

PTSD	Black or African American	3	8.94 [5.70-14.01]	<20	409	1.01 [0.6-1.72]	0.96
PTSD	White	3	8.81 [6.64-11.69]	48	1111	NA	
PTSD	Black or African American	4	5.11 [3.39-7.68]	23	578	0.66 [0.35-1.25]	0.21
PTSD	White	4	7.71 [4.73-12.59]	<20	430	NA	
SLEEP	Black or African American	1	49.37 [37.62-64.79]	52	167	1.49 [1.11-2]	0.010
SLEEP	White	1	33.04 [29.60-36.87]	318	1313	NA	
SLEEP	Black or African American	2	25.52 [20.87-31.20]	95	463	0.85 [0.68-1.06]	0.15
SLEEP	White	2	29.92 [27.25-32.85]	440	1577	NA	
SLEEP	Black or African American	3	32.53 [24.91-42.47]	54	337	0.82 [0.6-1.13]	0.22
SLEEP	White	3	39.43 [33.53-46.38]	146	781	NA	
SLEEP	Black or African American	4	17.83 [14.16-22.47]	72	521	0.48 [0.34-0.69]	<.001
SLEEP	White	4	36.82 [28.33-47.84]	56	320	NA	
SUD	Black or African American	1	27.76 [18.45-41.78]	23	123	2.15 [1.38-3.33]	0.002
SUD	White	1	12.94 [11.01-15.21]	147	1495	NA	
SUD	Black or African American	2	37.60 [29.89-47.29]	73	248	2.54 [1.95-3.3]	<.001
SUD	White	2	14.82 [13.00-16.90]	223	1610	NA	
SUD	Black or African American	3	36.15 [25.83-50.60]	34	187	1.96 [1.31-2.93]	0.002
SUD	White	3	18.46 [14.81-23.02]	79	841	NA	
SUD	Black or African American	4	43.81 [36.31-52.86]	109	357	1.98 [1.37-2.88]	<.001
SUD	White	4	22.11 [16.02-30.51]	37	338	NA	
SUIC	Black or African American	1	5.92 [3.08-11.37]	<20	209	2.22 [1.07-4.58]	0.048
SUIC	White	1	2.67 [1.95-3.65]	39	1869	NA	
SUIC	Black or African American	2	8.32 [6.03-11.49]	37	530	2.25 [1.52-3.32]	<.001
SUIC	White	2	3.71 [2.96-4.63]	77	2076	NA	
SUIC	Black or African American	3	17.63 [12.60-24.67]	34	375	2.33 [1.49-3.66]	<.001
SUIC	White	3	7.55 [5.60-10.18]	43	1140	NA	
SUIC	Black or African American	4	3.35 [2.02-5.56]	<20	565	0.47 [0.23-0.96]	0.039
SUIC	White	4	7.08 [4.34-11.56]	<20	455	NA	
DEM	Black or African American	1	6.05 [3.25-11.23]	<20	225	0.95 [0.5-1.84]	0.89
DEM	White	1	6.33 [5.14-7.79]	89	1851	NA	
DEM	Black or African American	2	4.15 [2.67-6.43]	20	564	1.01 [0.62-1.64]	0.97
DEM	White	2	4.11 [3.32-5.08]	85	2077	NA	
DEM	Black or African American	3	6.41 [3.80-10.83]	<20	418	0.89 [0.48-1.63]	0.70
DEM	White	3	7.22 [5.34-9.77]	42	1167	NA	
DEM	Black or African American	4	4.11 [2.62-6.45]	<20	586	0.56 [0.29-1.09]	0.095

© 2025 Wroblewski TH et al. *JAMA Network Open*.

DEM	White	4	7.31 [4.48-11.94]	<20	449	NA	
HACHE	Black or African American	1	27.26 [19.58-37.97]	35	171	1.26 [0.88-1.8]	0.21
HACHE	White	1	21.59 [18.96-24.59]	227	1424	NA	
HACHE	Black or African American	2	23.01 [18.67-28.36]	88	458	1.17 [0.92-1.48]	0.21
HACHE	White	2	19.74 [17.66-22.07]	309	1594	NA	
HACHE	Black or African American	3	52.50 [42.22-65.27]	81	310	1.87 [1.41-2.49]	<.001
HACHE	White	3	28.06 [23.35-33.71]	114	832	NA	
HACHE	Black or African American	4	25.40 [20.84-30.96]	98	507	1.05 [0.73-1.51]	0.78
HACHE	White	4	24.15 [17.85-32.68]	42	349	NA	
SEIZ	Black or African American	1	8.29 [4.82-14.28]	<20	212	1.43 [0.79-2.57]	0.26
SEIZ	White	1	5.81 [4.66-7.26]	78	1756	NA	
SEIZ	Black or African American	2	8.64 [6.29-11.87]	38	530	1.93 [1.32-2.83]	0.001
SEIZ	White	2	4.47 [3.63-5.51]	88	1949	NA	
SEIZ	Black or African American	3	10.67 [6.96-16.37]	21	377	1.25 [0.74-2.09]	0.41
SEIZ	White	3	8.56 [6.39-11.46]	45	1046	NA	
SEIZ	Black or African American	4	8.60 [6.14-12.03]	34	511	0.97 [0.55-1.72]	0.92
SEIZ	White	4	8.86 [5.58-14.06]	<20	417	NA	

© 2025 Wroblewski TH et al. *JAMA Network Open*.

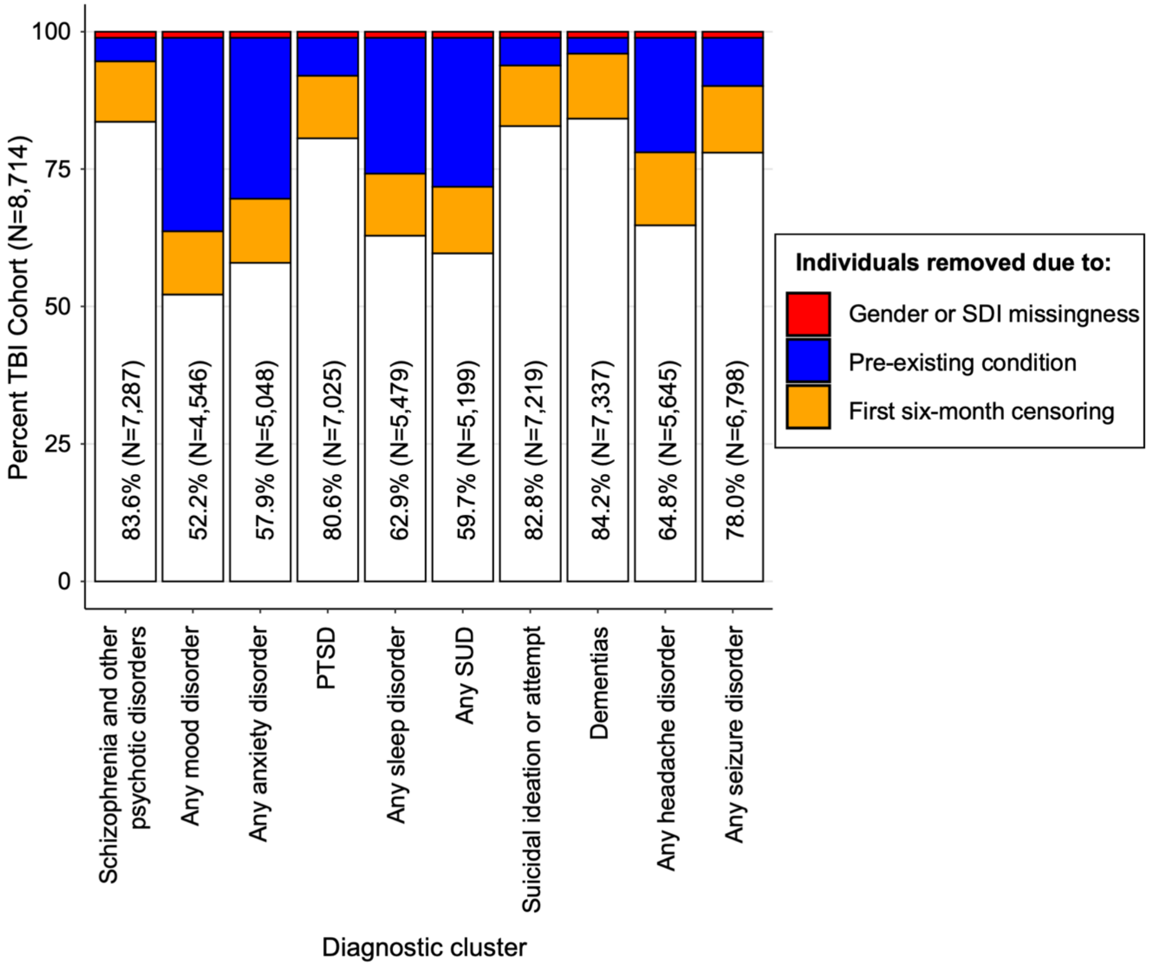

eFigure 1. The Proportion of Participants in the TBI Cohort (n = 8714) Included for Each Neuropsychiatric Disorder (NPD) Analysis. For each NPD (condition N_i), the total number of participants at risk (no-fill portion of the bar) represents a subset of the overall TBI cohort. To establish the analytical sample for each NPD, participants with missing gender or SDI data (red), a pre-existing diagnosis (blue) or insufficient EHR data (<6 months, orange) were excluded. The final analytical sample for each NPD is represented by the white portion of the bar accompanied by the percent of overall cohort and participant count.

© 2025 Wroblewski TH et al. *JAMA Network Open*.

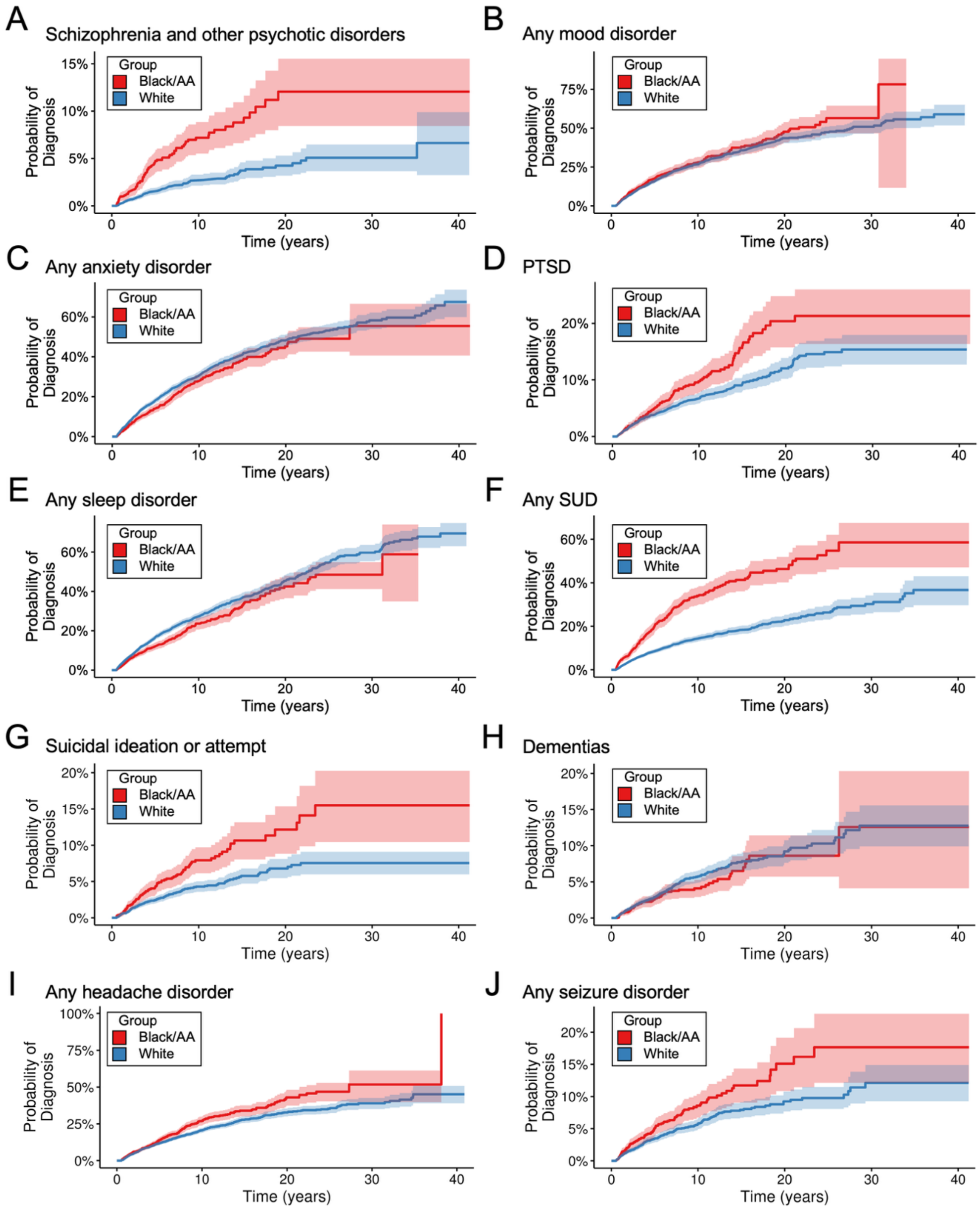

eFigure 2. Kaplan-Meier Curves Stratified by Racial Group. Curves represent the longitudinal probability of diagnosis for each neuropsychiatric diagnosis cluster (A-J) stratified by Black or African American (Black/AA) and White race groups.

© 2025 Wroblewski TH et al. *JAMA Network Open*.

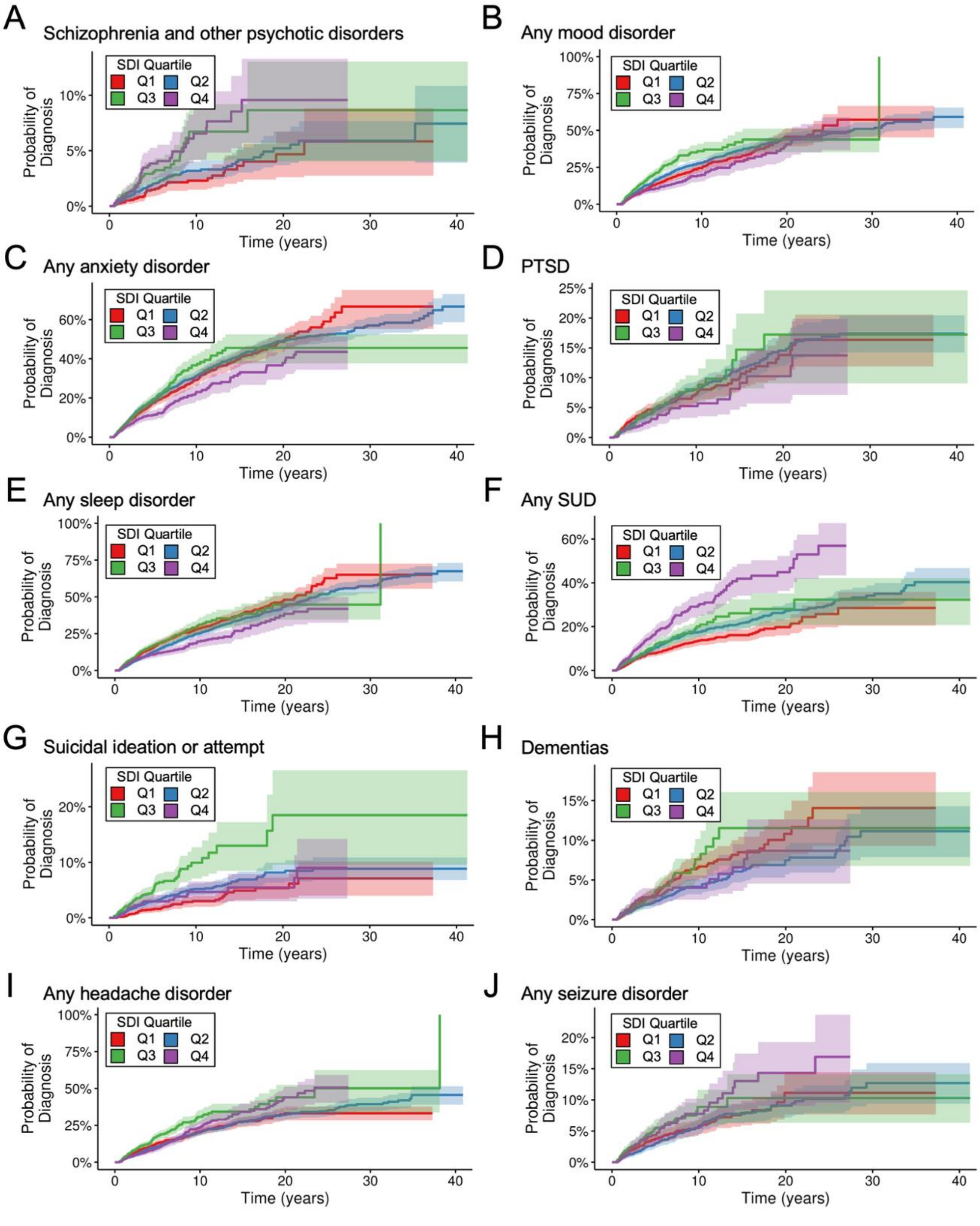

eFigure 3. Kaplan-Meier Curves Stratified by Social Deprivation Index (SDI) Quartile. Curves represent the longitudinal probability of diagnosis for each neuropsychiatric diagnosis cluster (A-J) assessed across SDI quartile. SDI is represented from the quartile with the lowest deprivation (Q1) through the quartile with the highest deprivation (Q4).

© 2025 Wroblewski TH et al. *JAMA Network Open*.

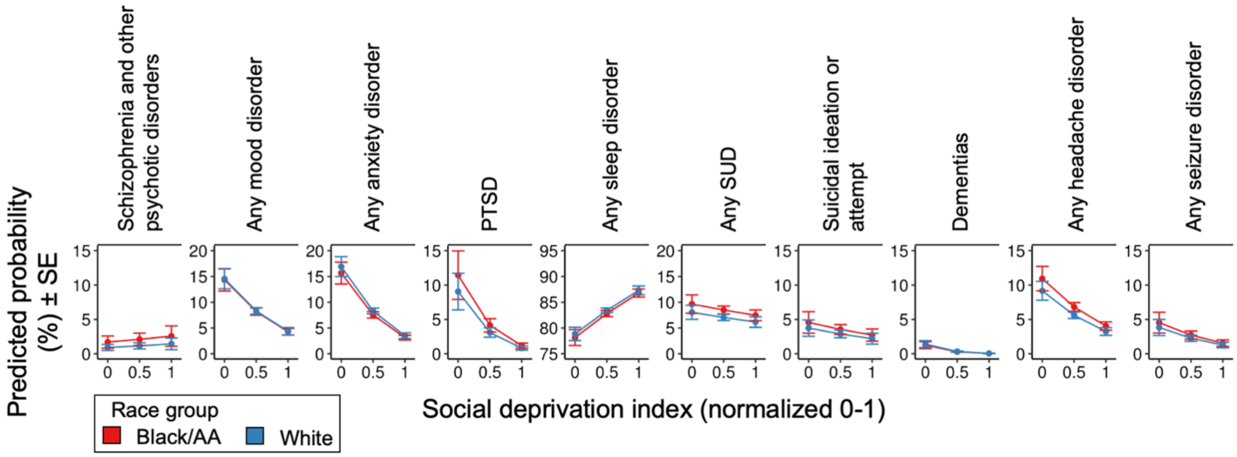

eFigure 4. Estimated Probabilities From Bivariate Probit Models (BPM). Displayed are predicted probabilities for BPMs for each neuropsychiatric diagnosis with all covariates set to reference and race covariable set to Black or African American (Black/AA; red) or White (blue); normalized social deprivation index (SDI) values set to 0, 0.5, and 1. *Y-axis* represents the predicted probability for each combination and error-bars represent the standard error.

© 2025 Wroblewski TH et al. *JAMA Network Open*.

eMethods

Heckman-Type Selection Model

Non-random selection of individuals into our cohort may be driven by a myriad of factors including disparities in access to healthcare and TBI diagnosis[1], exclusion of marginalized groups from research agendas[2,3], and pre-existing conditions that may both increase the risk for TBI and impact an individual's decision to participate in the AoU Program[4–6]. One approach to attempt to account for sample selection bias is through a Heckman-type selection model[7,8]. Heckman-type selection models are variants of the Heckman's two-step selection model originally developed to detect and statistically correct for selection bias in econometric models[9,10]. The original Heckman two-step model can only be implemented for continuous outcome variables[11,12]; therefore, this study adopts a bivariate probit model with sample selection (BPM) to estimate our dichotomous outcome variable[7,8,13–16]. This model estimates sample selection bias by simultaneously estimating two regression models, including a selection equation and an outcome equation, in a bivariate probit framework estimated by penalized maximum likelihood[8,14,17,18].

First, the selection model describes the selection into the TBI cohort (both as TBI diagnosis and participation in the AoU program) by estimating unobservable factors that may impact both the NPD outcome of interest as well as the likelihood of selection using a probit model:

$$t_i^* = \psi x_i + \phi z_i + \mu_i \quad (1)$$

$$t_i = \begin{cases} 1 \text{ if } t_i^* > 0 \\ 0 \text{ otherwise} \end{cases} \quad (2)$$

where t_i^* is the unobserved latent variable estimating the likelihood of TBI diagnosis (i.e., selection into the model) and depends on comprehensive adjustment of observed covariates x_i, the exclusion restriction z_i, and random error μ_i. t_i indicates actual TBI diagnosis status where 1 represents an established diagnosis and 0 indicates no recorded diagnosis. The exclusion restriction z_i is an observed variable included to prevent collinearity between the two models by modifying the selection equation but not serving as a relevant predictor of the outcome equation[7,8,13,19]. In this study, we argue that the presence of pre-existing neuropsychiatric diagnosis N_{pre} prior to index TBI serves as an appropriate exclusion restriction. Neuropsychiatric conditions prior to TBI have been previously shown to be a risk factor for sustaining a TBI[4–6], and may also impact participation in research programs, like AoU, as evidenced by lower prevalences of NPDs in the AoU Research Program compared to the general population[20]. Additionally,

© 2025 Wroblewski TH et al. *JAMA Network Open*.

individuals with a diagnosis of condition N_{pre} prior to index TBI cannot, by definition, have an incident diagnosis following TBI. Therefore, by the proposed model construction, N_{pre} would not serve as direct predictor of post-TBI N_i incidence, and instead, a sole predictor of selection into the cohort. To model selection into the TBI group, an additional control cohort (N=28,930) without a TBI diagnosis was established by randomly sampling 40,000 participants who were not included in the initial query for TBI diagnosis. Demographic information extraction, NPD diagnoses assignment, and filtering by Black or African American or White race group were performed using the same methods as in the case group [eTable 3].

The second equation is the outcome model, which like logistic regression models above, estimates the likelihood of individual i being diagnosed with condition N following TBI diagnosis

$$N_i^* = \beta x_i^* + \varepsilon_i \quad (3)$$

$$N_i = \begin{cases} 1 \text{ if } N_i^* > 0 \\ 0 \text{ otherwise} \end{cases} \quad (4)$$

where N_i^* is the unobserved latent variable estimating the likelihood of post-TBI neuropsychiatric diagnosis, which depends on observed covariates significantly associated with the N_i in univariable analysis (x_i^*) and random error (ε_i)[7,8,21]. N_i represents whether an individual was diagnosed with the condition N following index TBI.

In the standard Heckman-type model, parameter ρ estimates the correlation between error terms of the selection model (μ_i) and outcome model (ε_i)[7,8,21]. However, this approach requires the assumption of bivariate normally distributed error terms, which may be a severe limitation because this cannot easily be tested[14]. Instead, in this study we implemented a copula-based approach which allows for different dependence structures to be assessed, with the copula model of best fit determined by Akaike information criterion (AIC), as previously described[14,22]. Prior to applying the bivariate probit model, the Legrange multiplier test (score test) was applied to assess the null hypothesis of exogeneity (absence of non-random error), and a significant result indicates the presence of non-random sample selection in a dataset[23,24]. Outcomes are reported as coefficient estimates with standard error (ß±SE). Multiple testing correction was performed with a Bonferroni correction with significance set to P<.005.

© 2025 Wroblewski TH et al. *JAMA Network Open*.

eReferences

1. Wallace JS, Mannix RC. Racial Disparities in Diagnosis of Concussion and Minor Head Trauma and Mechanism of Injury in Pediatric Patients Visiting the Emergency Department. *The Journal of Pediatrics*. 2021;233:249-254.e1. doi:10.1016/j.jpeds.2021.01.057

2. Bailey ZD, Krieger N, Agenor M, Graves J, Linos N, Bassett MT. Structural racism and health inequities in the USA: evidence and interventions. *Lancet*. 2017;389(10077):1453-1463. doi:10.1016/S0140-6736(17)30569-X

3. Braveman PA, Arkin E, Proctor D, Kauh T, Holm N. Systemic And Structural Racism: Definitions, Examples, Health Damages, And Approaches To Dismantling. *Health Aff (Millwood)*. 2022;41(2):171-178. doi:10.1377/hlthaff.2021.01394

4. Huang MF, Su CH, Tu HP, et al. Association between bipolar disorder and subsequent traumatic brain injury in patients who received inpatient treatment. *Psychiatry Research*. 2018;261:517-521. doi:10.1016/j.psychres.2017.12.061

5. Deighton S, Buchy L, Cadenhead KS, et al. Traumatic brain injury in individuals at clinical high risk for psychosis. *Schizophrenia Research*. 2016;174(1):77-81. doi:10.1016/j.schres.2016.04.041

6. Vassallo JL, Proctor-Weber Z, Lebowitz BK, Curtiss G, Vanderploeg RD. Psychiatric risk factors for traumatic brain injury. *Brain Inj*. 2007;21(6):567-573. doi:10.1080/02699050701426832

7. Morrissey K, Kinderman P, Pontin E, Tai S, Schwannauer M. Web based health surveys: Using a Two Step Heckman model to examine their potential for population health analysis. *Social Science & Medicine*. 2016;163:45-53. doi:10.1016/j.socscimed.2016.06.053

8. Bärnighausen T, Bor J, Wandira-Kazibwe S, Canning D. Correcting HIV Prevalence Estimates for Survey Nonparticipation Using Heckman-type Selection Models. *Epidemiology*. 2011;22(1):27. doi:10.1097/EDE.0b013e3181ffa201

9. Heckman JJ. Sample Selection Bias as a Specification Error. *Econometrica*. 1979;47(1):153-161. doi:10.2307/1912352

10. Greene WH. Sample Selection Bias as a Specification Error: A Comment. *Econometrica*. 1981;49(3):795-798. doi:10.2307/1911523

11. Galimard JE, Chevret S, Curis E, Resche-Rigon M. Heckman imputation models for binary or continuous MNAR outcomes and MAR predictors. *BMC Med Res Methodol*. 2018;18:90. doi:10.1186/s12874-018-0547-1

12. Greene WH. *Econometric Analysis*. Pearson Education, Limited; 2012.

13. Bushway S, Johnson BD, Slocum LA. Is the Magic Still There? The Use of the Heckman Two-Step Correction for Selection Bias in Criminology. *J Quant Criminol*. 2007;23(2):151-178. doi:10.1007/s10940-007-9024-4

© 2025 Wroblewski TH et al. *JAMA Network Open*.

14. McGovern ME, Marra G, Radice R, Canning D, Newell ML, Bärnighausen T. Adjusting HIV prevalence estimates for non-participation: an application to demographic surveillance. *J Int AIDS Soc*. 2015;18(1):19954. doi:10.7448/IAS.18.1.19954

15. Marra G, Radice R, Bärnighausen T, Wood SN, McGovern ME. A Simultaneous Equation Approach to Estimating HIV Prevalence With Nonignorable Missing Responses. *Journal of the American Statistical Association*. 2017;112(518):484-496. doi:10.1080/01621459.2016.1224713

16. Marra G, Radice R. A joint regression modeling framework for analyzing bivariate binary data in R. *Dependence Modeling*. 2017;5(1):268-294. doi:10.1515/demo-2017-0016

17. Marra, Giampiero R Rosalba. GJRM: Generalised Joint Regression Modelling. Published online 2023:R package version 0.2-6.4.

18. Dubin JA, Rivers D. Selection Bias in Linear Regression, Logit and Probit Models. *Sociological Methods & Research*. 1989;18(2-3):360-390. doi:10.1177/0049124189018002006

19. Sartori AE. An Estimator for Some Binary-Outcome Selection Models Without Exclusion Restrictions. *Political Analysis*. 2003;11(2):111-138. doi:10.1093/pan/mpg001

20. Barr PB, Bigdeli TB, Meyers JL. Prevalence, Comorbidity, and Sociodemographic Correlates of Psychiatric Diagnoses Reported in the All of Us Research Program. *JAMA Psychiatry*. 2022;79(6):622. doi:10.1001/jamapsychiatry.2022.0685

21. Clark SJ, Houle B. Validation, Replication, and Sensitivity Testing of Heckman-Type Selection Models to Adjust Estimates of HIV Prevalence. *PLOS ONE*. 2014;9(11):e112563. doi:10.1371/journal.pone.0112563

22. Hasebe T. Copula-Based Maximum-Likelihood Estimation of Sample-Selection Models. *The Stata Journal*. 2013;13(3):547-573. doi:10.1177/1536867X1301300307

23. Marra G, Radice R, Filippou P. Regression spline bivariate probit models: A practical approach to testing for exogeneity. *Communications in Statistics - Simulation and Computation*. 2017;46(3):2283-2298. doi:10.1080/03610918.2015.1041974

24. Marra G, Radice R, Missiroli S. Testing the hypothesis of absence of unobserved confounding in semiparametric bivariate probit models. *Comput Stat*. 2014;29(3):715-741. doi:10.1007/s00180-013-0458-x

© 2025 Wroblewski TH et al. *JAMA Network Open*.
