# Supplementary material for: Inequities in Neuropsychiatric Outcomes After Brain Trauma in the All of Us Database
Source: JAMA Netw Open. 2025 Oct 24;8(10):e2539313. doi: 10.1001/jamanetworkopen.2025.39313 (PMC12552927; doi:10.1001/jamanetworkopen.2025.39313)
Supplement: Supplement 2. — Data Sharing Statement [file jamanetwopen-e2539313-s002.pdf]

Data Sharing Statement

Data
Data available: Yes
Data types: Deidentified participant data, Participant data with identifiers
How to access data: This study used data from the All of Us Research Program's Controlled Tier Dataset [v7], available to researchers who complete training through the All of Us Research Program and can be accessed through the Researcher Workbench (https://www.researchallofus.org/).
When available: With publication

Supporting Documents
Document types: Statistical/analytic code
How to access documents: Analytical code is maintained in the AoU Researcher Workbench (ID: aou-rw-dd4bca28), and can be made available upon reasonable request to corresponding author by email.
When available: With publication

Additional Information
Who can access the data: Data is available to researchers who complete training through the All of Us Research Program (https://www.researchallofus.org/).
Types of analyses: Information is available through the All of Us Research Program (https://www.researchallofus.org/). The workspace used for the analysis presented in this study can be found in the Research Project Directory (https://www.researchallofus.org/research-project-directory/) and is named "Biomarkers associated with TBI outcome in African ancestry individuals (v7)."
Mechanisms of data availability: Information is available through the All of Us Research Program (https://www.researchallofus.org/).
